# Primary Sjögren's syndrome with multiple calcifications in parotid glands

**DOI:** 10.1002/ccr3.2161

**Published:** 2019-10-13

**Authors:** Keisuke Kondo, Takahiro Kaneko, Norio Horie

**Affiliations:** ^1^ Department of Oral and Maxillofacial Surgery, Saitama Medical Center Saitama Medical University Saitama Japan

**Keywords:** multiple calcifications, parotid glands, Sjögren's syndrome

## Abstract

Dispersed calcifications in bilateral parotid glands may strongly suggest Sjögren's syndrome (SS), since the occurrence of bilateral calcifications in other salivary gland diseases is rare. This association between parotid calcifications and SS would thus represent highly useful information for the diagnosis of SS.

## Graphical abstract

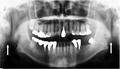

## 1 INTRODUCTION

Sjögren's syndrome (SS) is a chronic autoimmune disease characterized by damage to and dysfunction of the exocrine glands, specifically the salivary and lacrimal glands, mediated by autoantibodies and lymphocytic infiltrates, resulting in dry eyes and dry mouth. SS is accompanied by systemic manifestations including arthritis, fatigue, hematological abnormalities, pulmonary, renal and peripheral nervous system involvements, and lymphoma.^1^ SS typically affects middle‐aged women, and the prevalence of primary SS is approximately 0.05%‐0.23% in the general population.^2^ Some recent reports have described the appearance of small, dispersed calcifications in the parotid parenchyma as a useful feature for diagnosing SS.^3^, ^4^ Patients with SS frequently develop dry mouth and may thus initially visit dental and oral surgery clinics. We present herein a case of primary SS in which the diagnosis was suggested by bilateral small calcifications in the parotid glands on orthopantomography (OPG).

## 2 CASE REPORT

**Figure 1:**
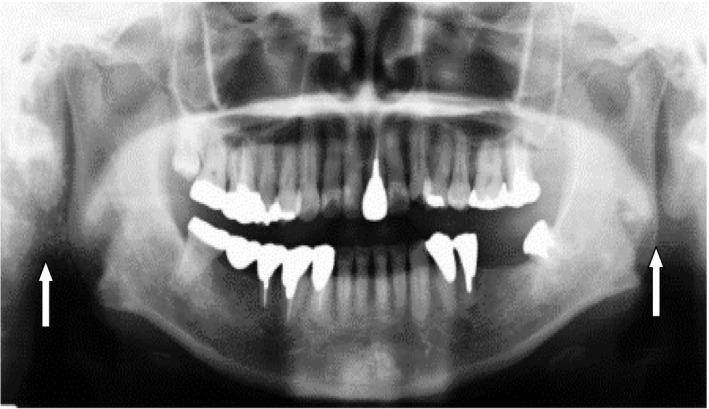
Orthopantomography shows multiple small calcifications around the right gonial angle and fewer calcifications around the left gonial angle

**Figure 2:**
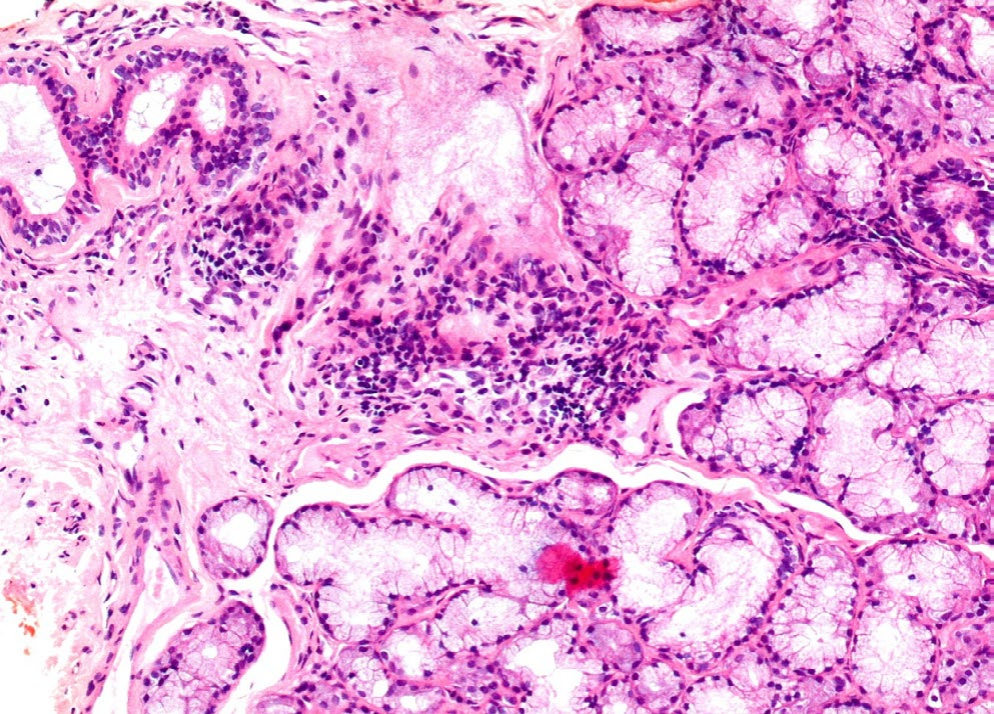
Photomicrograph with hematoxylin and eosin staining shows slight lymphocytic infiltrations around the ducts and slight fibrosis of the acini (original magnification 100×)

**Figure 3:**
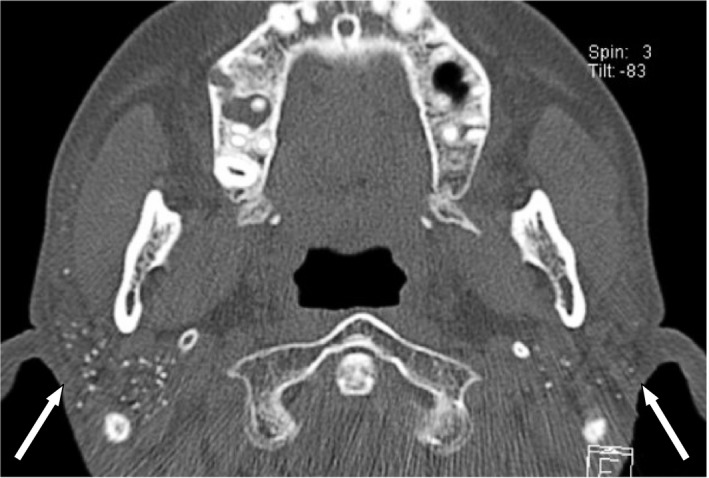
Axial computed tomography shows multiple small calcifications in bilateral parotid glands

A 49‐year‐old woman visited our clinic for detailed examination of dispersed calcifications in both parotid glands. The patient had been receiving treatment for dental caries, and her family dentist incidentally found calcifications on OPG. The patient had no medical history of note and was not taking any medications. Extraoral examination found no swelling or tenderness of the parotid glands or cervical lymphadenopathy and had no complaints of dry mouth or dry eyes. On oral examination, the oral mucosa was relatively dry and salivary flow from the parotid papilla was slight. On radiological examination by OPG, multiple small, dispersed calcifications were found in both parotid glands (Figure 1). The unstimulated whole saliva (UWS) flow rate was 0.30 mL/min (UWS flow rate in SS, 0.36 ± 0.33 mL/min based on the data of Márton et al^7^). Serum examination showed an increase in anti‐Ro/SS‐A (121 U/mL; positive, >10 U/mL), as the most specific autoantibody for SS. As a result, SS was strongly suspected, and the patient consulted the Rheumatology and Clinical Immunology Department of Internal Medicine. The specimen from a subsequent lip biopsy showed slight lymphocytic infiltrations around the ducts and slight fibrosis of the acini (Grade 0, Chisholm and Masson in 1968^8^; Figure 2). On computed tomography (CT), parotid glands on both sides showed fat replacement of the parenchyma and multiple small, dispersed calcifications in the parenchyma (Figure 3). The definite diagnosis was primary SS.

## 3 DISCUSSION

Patients with SS treated in departments of internal medicine rarely undergo routine imaging examinations of the parotid glands, with the exception of scintigraphy. However, patients who visit authorities with clinicians with dry mouth often undergo various image examinations of the parotid gland for the differential diagnosis of SS. For imaging, sonography, scintigraphy, and sialography are frequently used, and these images provide good depictions of the destruction of parotid gland parenchyma.

Recently, the occurrence of small, round calcifications in the parenchyma of the parotid gland, rather than the parotid duct, has gained attention as a potentially effective indicator of SS.^3^, ^4^ The frequency of these calcifications on CT has been retrospectively calculated as 29.4%‐46%, with bilateral occurrences in 23%‐35.2%.^3^, ^4^ The diagnostic effectiveness of identifying these calcifications has not been recognized, because parotid CT is not routinely performed for patients with SS. Little relationship has been found between the occurrence of calcifications and clinical symptoms, and the severity of parenchymal destruction.^3^ In some cases, the calcifications are clearly evident in OPG, but others may be missed due to their small size.

Whether these calcifications represent sialoliths or other structures such as angioliths remains unclear, but the typical features of sialoliths are lacking.^3^

Patients with SS frequently visit private dental clinics, because one of the major symptoms of SS is dry mouth. When a patient with dry mouth visits a clinic, the clinician may try to detect new SS by measuring the UWS flow rate^7^ and performing OPG, both of which are easily performed in dental clinics. In particular, disperse calcifications in bilateral parotid glands may strongly suggest SS, since the occurrence of bilateral calcifications in other salivary gland diseases is rare.^4^, ^5^ This association between parotid calcifications and SS would thus represent highly useful information for dental clinicians. OPG is imaging technology presented in most dental clinics can be a useful tool to aid in the diagnosis of Sjögren's syndrome.

## CONFLICT OF INTEREST

None declared.

## AUTHOR CONTRIBUTION

KK: designed the study, data collection, and wrote the initial draft of the manuscript. KK, TK and NH: authors have contributed to interpretation and critically reviewed the manuscript. NH: approved the final version of the manuscript.
